# Targeting of Immune Cells by Dual TLR2/7 Ligands Suppresses Features of Allergic Th2 Immune Responses in Mice

**DOI:** 10.1155/2017/7983217

**Published:** 2017-10-24

**Authors:** Jonathan Laiño, Andrea Wangorsch, Frank Blanco, Sonja Wolfheimer, Maren Krause, Adam Flaczyk, Tobias-Maximilian Möller, Mindy Tsai, Stephen Galli, Stefan Vieths, Masako Toda, Stephan Scheurer, Stefan Schülke

**Affiliations:** ^1^Vice President's Research Group 1: Molecular Allergology, Paul-Ehrlich-Institut, Langen, Germany; ^2^Department of Pathology and The Sean N. Parker Center for Allergy and Asthma Research, Stanford University School of Medicine, Stanford, CA, USA; ^3^Department of Microbiology and Immunology, Stanford University School of Medicine, Stanford, CA, USA

## Abstract

**Background:**

TLR ligands can promote Th1-biased immune responses, mimicking potent stimuli of viruses and bacteria.

**Aim:**

To investigate the adjuvant properties of dual TLR2/7 ligands compared to those of the mixture of both single ligands.

**Methods:**

Dual TLR2/7 ligands: CL401, CL413, and CL531, including CL264 (TLR7-ligand) and Pam_2_CysK_4_ (TLR2-ligand), were used. Immune-modulatory capacity of the dual ligands with the individual ligands alone or as a mixture in mouse BMmDCs, BMmDC:TC cocultures, or BMCMCs was compared and assessed in naïve mice and in a mouse model of OVA-induced intestinal allergy.

**Results:**

CL413 and CL531 induced BMmDC-derived IL-10 secretion, suppressed rOVA-induced IL-5 secretion from OVA-specific DO11.10 CD4^+^ TCs, and induced proinflammatory cytokine secretion *in vivo*. In contrast, CL401 induced considerably less IL-10 secretion and led to IL-17A production in BMmDC:TC cocultures, but not BMCMC IL-6 secretion, or IL-6 or TNF-*α* production *in vivo*. No immune-modulating effects were observed with single ligands. All dual TLR2/7 ligands suppressed DNP-induced IgE-and-Ag-specific mast cell degranulation. Compared to vaccination with OVA, vaccination with the mixture CL531 and OVA, significantly suppressed OVA-specific IgE production in the intestinal allergy model.

**Conclusions:**

Based on beneficial immune-modulating properties, CL413 and CL531 may have utility as potential adjuvants for allergy treatment.

## 1. Introduction

Th1-promoting adjuvants are promising candidates to improve the efficacy of allergy treatments. Studies have shown that exposure to bacterial or viral infections during early childhood may reduce the risk for the development of allergies later in life “hygiene hypothesis” [1]. Therefore, virus- or bacteria-derived PAMPs (pathogen-associated molecular patterns) which are able to shift an allergy-causing Th2 immune response towards a more tolerant Th1/Tr1-dominated phenotype are being investigated as vaccine components for improved allergy treatment.

In this context, TLR7 ligands are especially promising since they induce robust Th1-biased immune responses [2]. Cream containing the TLR7 ligand Imiquimod was shown to have a low irritating potential upon skin application and is undergoing extensive clinical testing as an adjuvant for topical treatment of human papillomavirus- (HPV-) induced warts, actinic keratosis, basal cell carcinoma, lentigo maligna, and molluscum contagiosum [2–4]. In the setting of basal cell carcinoma, Imiquimod induced both cytotoxic T cell responses and a pronounced proinflammatory cytokine secretion (TNF-*α*, IFN-*α*, IL-6, and IL-12) [5]. Coapplication of Imiquimod also was found to enhance antitumor responses in various mouse models [2]. However, Imiquimod treatment can result in toxic and inflammatory systemic symptoms such as fatigue, fever, malaise, pain, headache, nausea, diarrhea, and influenza-like symptoms [6]. Furthermore, studies have indicated a limited bioavailability of TLR7 ligands due to the rapid extracellular and intracellular degradation of purified TLR7 ligands by RNAses [7]. Therefore, intracellular TLR7 activation by purified TLR7 ligands in the target cell compartment is restricted. To circumvent this toxicity and reduced bioavailability, new approaches need to be developed that improve the efficacy of TLR7 ligand-based adjuvants that include improving their stability and their delivery inside the cell of interest.

In contrast to this, bacterial lipopeptides such as the TLR2/6 ligand Pam_2_CysK_4_ have been shown to be stable and potent adjuvants that induce tolerogenic DC and regulatory T cell responses [8, 9]. Moreover, they can induce a Th1-promoting cytokine milieu and enhance Ag presentation of endogenous peptides [10, 11]. Chemical conjugation of the TLR2 ligand Pam_3_CysK_4_ to OVA-derived CD8^+^ TC peptide sequences resulted in a rapid and enhanced uptake in DCs [12]. Additionally, TLR2 stimulation of mouse Th1 cells induced IFN-*γ* production, cell proliferation, and cell survival without additional TCR stimulation [13], while IL-5, IL-13, and IFN-*γ* responses in cells derived from human house dust mite-allergic patients were inhibited by Pam_3_CysK_4_ [14]. These properties suggest that TLR2/6 ligands may have utility in the treatment of allergies.

**Figure 1:**
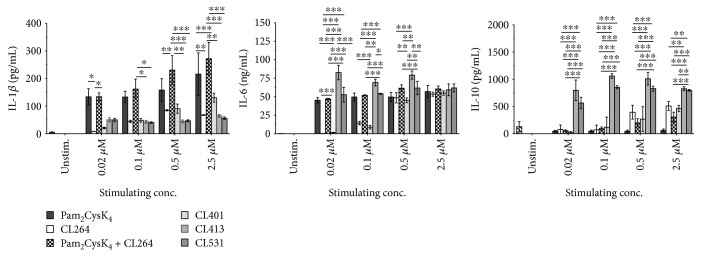
The dual TLR2/7 ligands CL413 and CL531 induce a strong BMmDC IL-10 secretion. Cytokine secretion from stimulated BALB/c BMmDCs. Data are mean results of three independent experiments ± SD. ^∗^*P* < 0.05; ^∗∗^*P* < 0.01; ^∗∗∗^*P* < 0.001.

In an attempt to improve the bioavailability and to leverage the Th1-inducing potential of TLR2 and TLR7 ligands, we investigated three different, commercially available, synthetic dual TLR2/7 ligands: CL401, CL413, and CL531. These dual TLR2/7 ligands contain CL264, a TLR7-activating 9-benzyl-8-hydroxyadenine which is conjugated to different positions of the TLR2/6 ligand Pam_2_CysK_4_: CL401 combines CL264 directly with Pam_2_Cys, whereas for CL413, and CL531, CL264 is conjugated to the terminal acid function or to the lateral chain of the second lysine of Pam_2_CysK_4_, respectively (Repository Figure 1 available online at https://doi.org/10.1155/2017/7983217) [15].

In a mouse B16 melanoma model, intratumoral administration of CL401 and CL413 into established tumors resulted in reduced tumor growth and enhanced survival [15]. Moreover, after the initial submission of the present manuscript, Gutjahr and coworkers recently published that coapplication of a similar dual TLR2/7 ligand PamadiFectin (CL307) and HIV-1 antigen p24-coated nanoparticles can efficiently boost HIV-specific antibody responses while also inducing a balanced Th1/Th2 profile in mice [16]. We hypothesized that chemical conjugation of different TLR ligands could be used to create dual TLR2/7 ligands which can promote TLR-mediated Th1-biased immune responses. This new class of adjuvants may be able to mimic the potent immune stimuli of viruses and bacteria and may facilitate penetration of the TLR7 ligand into the cell by TLR2-mediated uptake and trafficking. However, there is currently very little data concerning the immune-activating properties of these novel adjuvants.

Here, we evaluated three different dual TLR2/7 ligands and compared their immune-modulating capacity to equimolar amounts of the two-component ligands, either tested alone or provided as a mixture. We analyzed their effects on mouse bone marrow-derived myeloid dendritic cells (BMmDCs), BMmDC:TC cocultures, and bone marrow-cultured mast cells (BMCMCs). Moreover, we also investigated their immune-activating potential in naïve mice as well as the immune-modulating effect of CL531 upon prophylactic vaccination in combination with OVA in a mouse model of OVA-induced intestinal allergy.

## 2. Materials and Methods

### 2.1. Mice

BALB/c, OVA-TCR transgenic DO11.10 (BALB/c background), and C57BL/6 mice (Jackson Laboratories, Bar Harbor, Maine, USA) were bred under specific pathogen-free conditions at the animal facilities of the Paul-Ehrlich-Institut and Stanford University Medical School, respectively.

### 2.2. Antigens and TLR Ligands

Recombinant ovalbumin (OVA) was produced according to Schülke et al. [17]. CL264, CL401, CL413, CL531 (all with an endotoxin level < 0.001 EU/*μ*g), and Pam_2_CysK_4_ (absence of endotoxins controlled by HEK-Blue™ TLR4 cells by the manufacturer) were purchased from InvivoGen (Toulouse, France).

### 2.3. Epithelial Cell Activation

Mouse lung epithelial cells (LA-4, ATTC® CCL-196) were cultured in DMEM (Lonza, Basel, Switzerland) containing L-glutamine (1 mM), penicillin (100 U/mL), streptomycin (100 *μ*g/mL), and 15% fetal calf serum (FCS, Sigma-Aldrich, Steinheim, Germany). For stimulation assays, cells were harvested, taken up in medium containing 2% FCS, and 6.5 × 10^5^ cells were cultivated in 24-well plates (Thermo Fisher Scientific, Langenselbold, Germany) at 37°C in a humidified atmosphere of 5% CO_2_ overnight. Nonadherent cells were removed by replacing the medium, and cells were stimulated with equimolar amounts of Pam_2_CysK_4_, CL264, Pam_2_CysK_4_ plus CL264, CL401, CL413, or CL531 in a total volume of 0.5 mL for 24 h. Levels of CCL2 were determined 24 h poststimulation using the CCL2 Ready-SET-Go! ELISA Kit (eBiosciences, Frankfurt, Germany).

### 2.4. *In Vitro* Generation of Mouse Bone Marrow-Derived Dendritic Cells

Mouse myeloid dendritic cells (referred to herein as BMmDCs) and plasmacytoid dendritic cells (referred to herein as BMpDCs) were generated as described previously [17, 18]. Briefly, bone marrow cells were isolated from the femurs and tibias of BALB/c mice and differentiated into BMmDCs using GM-CSF or into BMpDC using Flt-3L (both R&D Systems, Minneapolis, USA) for eight days.

### 2.5. Dendritic Cell Activation

BALB/c BMmDCs or BMpDCs were seeded at 3.2 × 10^5^ cells/mL in 24-well plates (Thermo Fisher Scientific) and stimulated with equimolar amounts of Pam_2_CysK_4_, CL264, Pam_2_CysK_4_ plus CL264, CL401, CL413, or CL531 for 24 h. Supernatants were analyzed for cytokine secretion by ELISA using BD Opteia ELISA sets (BD Biosciences, Heidelberg, Germany).

### 2.6. Preparation of BMmDC and CD4^+^ T Cell Cocultures

Splenic CD4^+^ T cells were isolated from OVA-TCR transgenic DO11.10 mice using the CD4 T Cell Isolation Kit (Miltenyi Biotec, Bergisch Gladbach, Germany). BALB/c-derived BMmDCs (3.2 × 10^5^ cells/mL) were either cultured alone or together with DO11.10 CD4^+^ T cells (8.0 × 10^5^ cells/mL, >95% purity) and stimulated with equimolar amounts of Pam_2_CysK_4_, CL264, Pam_2_CysK_4_ plus CL264, CL401, CL413, or CL531 in the presence or absence of 20 *μ*g/mL rOVA for 72 h. Subsequently, concentrations of IL-1*β*, IL-2, IL-4, IL-5, IL-6, IL-10, IL-13, IL-17A, TNF-*α*, and IFN-*γ* in the supernatants were measured by either BD OptEIA™ ELISA (BD Biosciences) or Ready-SET-Go! ELISA Sets (eBiosciences).

### 2.7. Mast Cell Generation and Functional Analysis

C57BL/6 and BALB/c bone marrow cells were differentiated into bone marrow-derived cultured mast cells (BMCMCs) by using 20% WEHI-3-conditioned, complete CMLESS medium (DMEM 10% FCS, 2 mM L-glutamine, 5 × 10^−5^ M *β*-mercaptoethanol, and 1% antibiotic/antimycotic) for 6 weeks. 1 × 10^6^ C57BL/6 BMCMCs were stimulated with the indicated equimolar amounts of Pam_2_CysK_4_, CL264, Pam_2_CysK_4_ plus CL264, CL401, CL413, or CL531 for 24 h in 24-well plates in a total volume of 1 mL complete CMLESS medium conditioned with 20% WEHI-3-supernatant. 50 ng/mL PMA plus 10 *μ*M A23187 calcium ionophore (both Sigma-Aldrich) was used as positive control. After 24 h, plates were centrifuged at 400 g at RT for 5 min and supernatants were removed and analyzed for IL-6 secretion by ELISA using the BD OptEIA IL-6 ELISA set according to the manufacturer's recommendations (BD Biosciences). BMCMCs were analyzed for cell viability by propidium iodide (Life Technologies, Carlsbad, CA) incorporation 24 h poststimulation.

To investigate the influence of the different TLR ligands on mast cell (MC) degranulation, C57.1 mouse MCs were maintained in complete CMLESS medium at a concentration of 5 × 10^5^ cells/mL. For degranulation assays, 2 × 10^5^ C57.1 MCs were sensitized with 2 *μ*g/mL anti-DNP IgE [19] overnight and stimulated with 10 ng/mL of DNP-HSA (30–40 mol DNP per mol albumin, Sigma-Aldrich) in the presence or absence of the indicated amounts of Pam_2_CysK_4_, CL264, Pam_2_CysK_4_ plus CL264, CL401, CL413, or CL531 for 1 h in a total volume of 50 *μ*L 1x Tyrode's buffer. 50 ng/mL PMA plus 10 *μ*M A23187 calcium ionophore was used as positive control (Sigma-Aldrich). Subsequently, 10 *μ*L of both cell supernatant and cell lysate was analyzed for *β*-hexosaminidase content using p-NAG (Sigma-Aldrich) as substrate. Secretion of *β*-hexosaminidase was calculated as % release = OD (sup.)/((OD_sup._ + OD_lysate_)/100).

### 2.8. Analysis of ERK1/2 Phosphorylation in BMCMCs

For detection of phosphorylated ERK1/2, C57BL/6-derived BMCMCs were stimulated with 50 ng/mL PMA plus 10 *μ*M A23187 calcium ionophore or 5 *μ*M of either Pam_2_CysK_4_, CL264, Pam_2_CysK_4_ plus CL264, CL401, CL413, or CL531 for 5 min at 37°C and 5% CO_2_. Additionally 5 × 10^5^ BMCMCs/mL were sensitized with 2 *μ*g/mL anti-DNP IgE [19] overnight and stimulated with 10 ng/mL DNP-HSA for 5 min at 37°C 5% CO_2_. Subsequently, cells were fixed with 4% PFA for 15 min, permeabilized with cold methanol for 30 min, and stained for CD117 (APC-conjugate, eBiosciences) and pERK1/2 using an AF647-conjugated anti-pERK1/2 (Thr202/Tyr204) antibody (Cell Signaling Technologies, Danvers, MA) for 2 h. For pERK1/2 analysis, BMCMC preparations were gated on CD117-positive mast cells.

### 2.9. *In Vivo* Administration of TLR Ligands

For measurements of cytokine secretion *in vivo*, BALB/c mice (*n* = 5 per group) were injected i.p. with 0.2 mM of the different ligands in a final volume of 0.2 mL PBS. Blood samples were drawn by cardiac puncture 8 h postinjection and collected in Z-gel tubes (Sarstedt, Nümbrecht, Germany). Levels of IL-1*β*, IL-6, IL-12p70, and TNF-*α* in mouse sera were measured using BD Opteia ELISA sets (BD Biosciences). Levels of IFN-*α* were measured using the VeriKine™ Mouse IFN Alpha ELISA Kit (pbl Assay Science, New Jersey, USA) according to the manufacturer's recommendations. All animal experiments were performed in compliance with approved protocols by the German Animal Protection Law (local approval number: F107/131) or Stanford University.

### 2.10. Prophylactic Vaccination in a Mouse Model of Intestinal Allergy

For prophylactic vaccination, BALB/c mice (female, 8–12 weeks) were vaccinated three times in three-day intervals by i.n. administration of the following substances (all in 30 *μ*l volume): (a) nontreated mice (Mock group)—received PBS as mock vaccination; (b) nonvaccinated allergic positive control group (PBS)—mice that received PBS; (c) OVA group (OVA)—10 *μ*g rOVA; (d) TLR2 ligand group (Pam_2_CysK_4_ + OVA)—0.5 mM Pam_2_CysK_4_ + 10 *μ*g rOVA; (e) TLR7 ligand group (CL264 + OVA)—0.5 mM CL264 + 10 *μ*g rOVA; (f) TLR2/7 dual-ligand group (CL531 + OVA)—0.5 mM CL531 + 10 *μ*g rOVA; and (g) TLR2/7 dual-ligand control (no OVA) group (CL531)—0.5 mM CL531 (for comparison of experimental groups see also Repository Figure 5).

One week after the last vaccination, mice from PBS, OVA, Pam_2_CysK_4_ + OVA, CL264 + OVA, CL531 + OVA, and CL531 groups were sensitized to OVA twice in a biweekly interval by i.p. injection of 10 *μ*g OVA (grade V) absorbed to 2 mg aluminum-hydroxide adjuvant (Pierce, Solingen, Germany) in 200 *μ*L sterile PBS (OVA/A). Mice in the Mock group received PBS as mock sensitization.

For induction of intestinal allergy, two weeks after the second sensitization, mice were challenged by being fed an egg white diet containing OVA for 8 days (PBS, OVA, Pam_2_CysK_4_ + OVA, CL264 + OVA, CL531 + OVA, and CL531 groups) or by being fed a conventional diet (PBS group) free from OVA as control [20]. Blood samples were collected after vaccination (day 14) and after sensitization (day 42) via the tail vein and on the final day of EW diet, by cardiac puncture under deep ketamine/rompun anaesthesia (Figure 5(a)). All animal experiments were performed in compliance with approved protocols by the German Animal Protection Law (local approval number: F107/131).

### 2.11. Detection of OVA-Specific IgG1, IgG2a, and IgE Levels in Mouse Sera and Detection of Mediator Release from RBL 2H3 Cells

For measurement of OVA-specific IgG1, IgG2a, and IgE antibody titers in mouse sera, ELISA plates (Greiner Bio-One, Solingen-Wald, Germany) were coated with 5 *μ*g/well OVA (OVA grade V, Sigma) in coating buffer (50 mM Na_2_CO_3_/NaHCO_3_, pH 9.6) overnight at 4°C. Serum samples (50 *μ*L/well) were diluted by serial dilution (for IgE: 1 × 1 : 10, then 6 × 1 : 5, for IgG1 and IgG2a: 1 × 1 : 100, then 6 × 1 : 10) and incubated at 4°C overnight (IgE) or for 2 h at room temperature (IgG1, IgG2a). Levels of OVA-specific antibodies were measured using 50 *μ*L secondary detection antibody diluted in PBS supplemented with 10% FCS (IgE: rat anti-mouse IgE biotin conjugated, BD Biosciences, Heidelberg, Germany 1 : 1000; IgG1: goat anti-mouse IgG1*γ*1 HRP-conjugated, 1 : 8000; and IgG2a: rabbit anti-mouse IgG2a, 1 : 8000—all Invitrogen, Darmstadt, Germany—incubation time: 1.5 h for IgG1 and IgG2a and overnight for IgE) in combination with a streptavidin-HRP antibody (for IgE detection, 50 *μ*L diluted 1 : 2000 in PBS supplemented with 10% FCS, BD Biosciences) applied for 30 min at room temperature. Development was performed with 100 *μ*L/well TMB substrate solution (BD Biosciences) for up to 30 min. The reaction was stopped by the addition of 50 *μ*L/well 25% sulfuric acid and analyzed using a SpectraMAX340PC (Molecular Devices, CA) reading the absorbance at 450 nm. Data were analyzed using Excel and Graphpad Prism (GraphPad Software, La Jolla, CA, USA). Measurement of mediator release from RBL-2H3 cells was performed by sensitizing RBL 2H3 cells with pooled sera and by quantifying of *β*-hexosaminidase release according to [20]. In short, 1.5 × 10^5^ RBL 2H3 cells/well were seeded in 96-well plates overnight. The next day, medium was removed by aspiration, pool sera were diluted 1 to 10 in medium, 50 *μ*L serum dilution per well were added to the cells in triplicates, and cells were sensitized for 1 h at 37°C. Subsequently, plates were washed with 1x Tyrode's buffer (137 mM NaCl, 2.7 mM KCl, 0.4 mM NaH_2_PO_4_, 0.5 mM MgCl_2_^∗^6H_2_O, 1 mM CaCl_2_, 0.1% BSA, 0.1% glucose, 10 mM HEPES, pH 7.45), 100 *μ*L of the indicated amounts of OVA (grade V, Sigma-Aldrich) in 1x Tyrode's buffer was added to the cells, and plates were incubated at 37°C. After one hour, 30 *μ*L of supernatant was transferred to a new 96-well plate and mixed with 50 *μ*L/well substrate solution (0.1 M pNAG, pH 4.5 in Na_2_HPO_4_). Plates were incubated for 45 minutes at 37°C and stopped with 100 *μ*L/well stop solution (0.2 M glycine, pH 10.7).

### 2.12. Statistical Analysis

Comparison between different treatment groups was performed by means of 2-way ANOVA analysis. Confidence intervals for the estimated differences between treatment groups as well as *p* values were adjusted using the Bonferroni method in order to restrict the overall type I error *α* (false-positive results, that is, false significant differences) to 5%. *p* values < 0.05, <0.01, and <0.001 were designated with ∗, ∗∗, and ∗∗∗, respectively. The statistical analyses were performed with GraphPad Prism 6 software for Mac, version 6.0f.

## 3. Results

### 3.1. The Dual TLR2/7 Ligands CL413 and CL531 Induce Strong Secretion of IL-10 by BMmDCs and Suppress rOVA-Induced Th2 Cytokine Secretion

**Figure 2:**
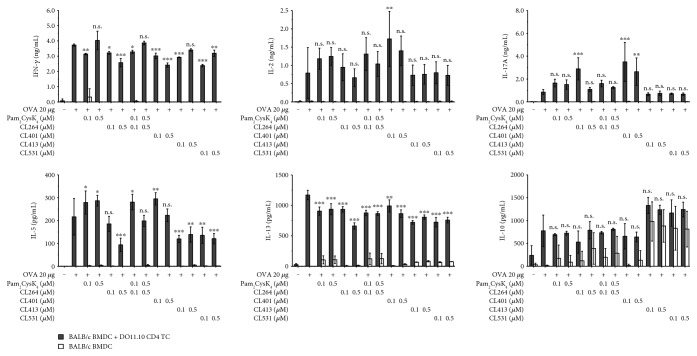
The dual TLR2/7 ligands CL413 and CL531 suppress rOVA-induced Th2 cytokine secretion while CL401 induces IL-17A secretion. Cytokine secretion from stimulated BALB/c BMmDC:DO11.10 CD4^+^ TC cocultures. Data are mean results of two independent experiments ± SD. n.s. /∗/∗∗/∗∗∗: statistical significance compared to the OVA 20 *μ*g group.

First, we checked the potential of the different TLR ligands to induce the activation of mouse bone marrow-derived myeloid dendritic cells (BMmDCs) since these cells are highly important in the establishment of adaptive immune responses when applying these adjuvants in a vaccination setting. When analyzing the activation profile of *in vitro* differentiated BMmDCs (Figure 1, gating strategy and phenotype shown in Repository Figure 2), we observed that, compared to Pam_2_CysK_4_ alone or mixed with CL264, the dual TLR2/7 ligands CL413 and CL531 resulted in significantly reduced IL-1*β* secretion, even when applied in higher concentrations. CL264 and CL401 dose-dependently induced IL-1*β* secretion, at lower levels than Pam_2_CysK_4_. In contrast, IL-6 secretion at low-stimulation doses (0.2 and 0.1 *μ*M) was similar among Pam_2_CysK_4_-, Pam_2_CysK_4_ + CL264-, and CL531-stimulated cells, whereas CL413 stimulation resulted in a significantly higher IL-6 secretion than the other ligands. For CL264 and CL401, at least 0.5 *μ*M of each ligand was required to induce levels of IL-6 secretion similar to those of the other dual TLR2/7 ligands. Most interestingly, only CL413 and CL531 induced a highly significant induction of the anti-inflammatory cytokine IL-10, especially notable at low concentrations of the agents.

**Figure 3:**
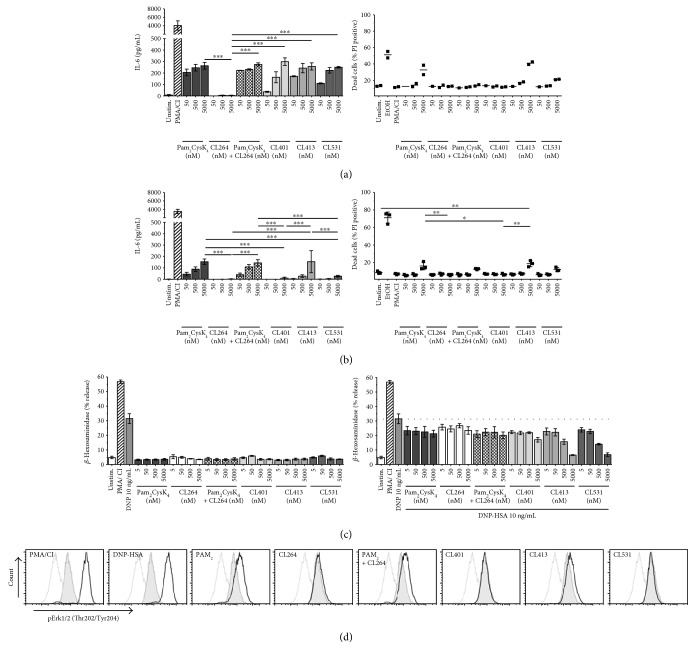
Dual TLR2/7 ligands induce limited mast cell activation directly but suppress IgE-and-antigen-induced mast cell degranulation. IL-6 secretion and cytotoxicity of stimulated BALB/c (a) and C57BL/6 (b) BMCMCs. TLR2/7 ligand-induced degranulation in the presence or absence of DNP-HSA from *α*DNP-IgE-sensitized C57.1 MCs. Dashed line indicates the level of *β*-hexosaminidase release induced by stimulation with 10 ng/mL DNP-HSA alone (c). Analysis of phospho-ERK1/2 (Thr202/Tyr204) levels in C57BL/6 BMCMCs stimulated with 5 *μ*M of the different TLR ligands for 5 min (d). Grey: unstained cells; grey tinted: unstimulated; black: stimulated as indicated; Data are representative results of two (d) or mean of three (a, b, c) independent experiments ± SD. ^∗^*P* < 0.05; ^∗∗^*P* < 0.01; ^∗∗∗^*P* < 0.001.

We also investigated cytokine secretion induced by the different ligands from Flt-3L-cultures containing approximately 10% bone marrow-derived plasmacytoid dendritic cells (BMpDCs) (Repository Figure 3, gating strategy and phenotype shown in Repository Figure 2). While dendritic cells within Flt-3L-cultures did not secrete high amounts of IL-1*β* upon stimulation with any of the tested ligands, we were able to detect high levels of IL-6 secretion upon stimulation with either Pam_2_CysK_4_, Pam_2_CysK_4_ + CL264, CL413, or CL531, but not CL264 or CL401 (Repository Figure 3). Here, in lower stimulation concentrations (0.1 and 0.5 *μ*M), CL413 and CL531 tended to induce slightly higher (although not significantly higher) levels of IL-10 production than Pam_2_Cysk_4_ and CL264 (Repository Figure 3).

CL413 and CL531 also enhanced BMmDC-derived IL-10 secretion in BMmDC:DO11.10 CD4^+^ TC coculture (Figure 2). Here, this IL-10 secretion did not reach statistical significance. While only slightly reducing rOVA-induced IFN-*γ* secretion, costimulation with CL413 or CL531 resulted in significantly reduced IL-13 and IL-5 levels compared to stimulation with rOVA alone (Figure 2). In contrast, CL401 had no effect on rOVA-induced IL-5 secretion, but induced significantly elevated levels of IL-17A (Figure 2).

**Figure 4:**
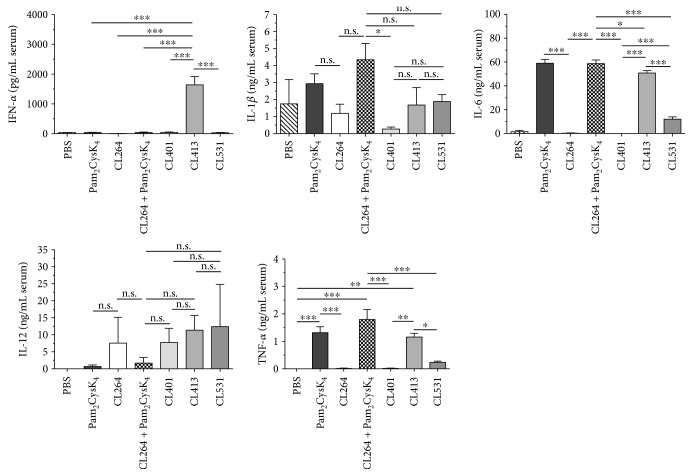
Dual TLR2/7 ligands induce both TLR2- and TLR7-mediated cytokine secretion *in vivo*. Serum cytokine levels 8 h post i.p. application of 0.2 mM of the indicated ligands. Data are mean results of 5 mice per group ± SD. ^∗^*P* < 0.05; ^∗∗^*P* < 0.01; ^∗∗∗^*P* < 0.001.

In mouse LA-4 epithelial cells, stimulation with Pam_2_CysK_4_ (with or without CL264), CL413, or CL531 induced similar levels of CCL2 production whereas CL401-induced CCL2 secretion was only observed at higher doses and CL264 was without detectable effect (Repository Figure 4).

In summary, these results suggest that the dual TLR2/7 ligands CL413 and CL531 might be of potential value as adjuvants for the treatment of allergic diseases because of their capacity to induce tolerogenic IL-10 secretion from BMmDCs and to suppress the secretion of Th2-cytokines from allergen-specific T cells.

### 3.2. Dual TLR2/7 Ligands Induce Limited Mast Cell Activation

Next, we evaluated the potential of the different ligands to induce direct mast cell activation and degranulation. Direct mast cell activation is a hallmark feature of allergic reactions and potential adjuvants for the treatment of allergies should neither induce mast cell degranulation by themselves nor enhance mast cell degranulation upon coapplication with an allergen. When stimulating BALB/c (Figure 3(a)) or C57BL/6 (Figure 3(b)) bone marrow-derived cultured mast cells (BMCMCs) with the different ligands, we observed a dose-dependent BMCMC activation with all ligands except CL264 in BALB/c-derived BMCMCs (Figure 3(a)), while IL-6 secretion was less pronounced for all activators in C57BL/6-derived BMCMCs (Figure 3(b)). We also observed initial toxic effects upon stimulation with the highest dose (5 *μ*M) of Pam_2_CysK_4_, CL413, and CL531 in BMCMCs derived from either strain (Figure 3(b)). In this experimental setting, for all tested concentrations, no upregulation of ICOS-L or OX-40L and no secretion of IL-4, IL-10, IL-12, or GM-CSF was detected in BALB/c or C57BL/6 BMCMCs stimulated with the different TLR2 and TLR7 ligands (data not shown).

Analysis of DNP-HSA-induced C57.1 mast cell degranulation revealed that, even when applied in high concentrations, all ligands induced very little direct C57.1 degranulation (Figure 3(c)). Notably, when coapplied with DNP-HSA, all dual TLR2/7 ligands dose-dependently suppressed DNP-HSA-induced degranulation. Remarkably, this effect was not observed for Pam_2_CysK_4_ and CL264 either provided alone or provided as a mixture (Figure 3(c)).

Mechanistically, compared to unstimulated controls, stimulation of BMCMCs with 5 *μ*M Pam_2_CysK_4_, Pam_2_CysK_4_ + CL264, or CL413, but not with CL264, CL401, or CL531, resulted in increased levels of ERK1/2 phosphorylation (Figure 3(d)), consistent with the induction of IL-6 secretion from BMCMCs by these ligands (Figure 3(b)). However, no induction of phosphoPLC*γ*1 was observed upon stimulation with the different ligands (data not shown).

### 3.3. Dual TLR2/7 Ligands Induce Both TLR2- and TLR7-Mediated Cytokine Secretion in Mice

Next, we evaluated the cytokine response induced by application of the different ligands *in vivo* in an established mouse model of TLR ligand-induced immune activation (Figure 4, [21]). For this purpose, we measured serum cytokine concentrations 8 h post i.p. application of the different ligands in BALB/c mice. Interestingly, of all TLR ligands tested, only CL413 resulted in a robust, and compared to the other groups, highly significant secretion of IFN-*α* (Figure 4). Moreover, we found that IL-6 production and TNF-*α* production were only induced in the presence of Pam_2_CysK_4_ (either alone or contained within the TLR2/7 ligands CL413 and CL513) but not CL264 (Figure 4). Remarkably, for both cytokines, CL401 did not induce significant secretion of these proinflammatory cytokines. Moreover, IL-1*β* secretion was only observed in animals injected with either Pam_2_CysK_4_ or Pam_2_CysK_4_ + CL264 (Figure 4). Of note, Th1-promoting IL-12 production was observed for ligands containing the TLR7 ligand CL264, either alone or as part of a dual TLR2/7 ligand (CL264, CL401, CL413, and CL531, Figure 4). We found that all dual TLR2/7 ligands induced similar levels of IL-12 production (Figure 4).

### 3.4. Prophylactic Vaccination with CL531 Suppresses OVA-Specific IgE Production in a Mouse Model of OVA-Induced Intestinal Allergy

**Figure 5:**
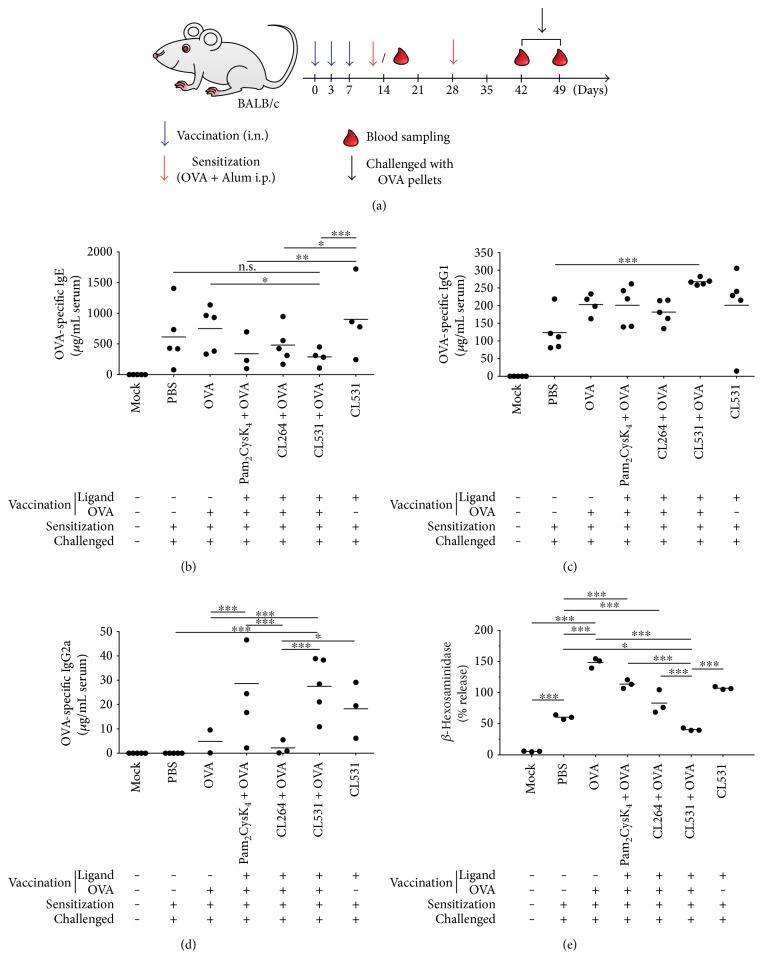
Prophylactic vaccination with CL531 plus OVA suppresses OVA-specific IgE production while inducing OVA-specific IgG production in a mouse model of OVA-induced intestinal allergy. Schematic representation of prophylactic vaccination approach (a). Blue arrows represent intranasal (i.n.) vaccination or mock vaccination (in “Mock” group); red arrows represent intraperitoneal (i.p.) sensitization with 10 *μ*g OVA with 2 mg Alum in 200 *μ*L sterile PBS (or, in “Mock” group, with sterile PBS alone). Blood drops represent blood sampling from the tail after vaccination and sensitization or cardiac puncture after challenged with OVA-containing food pellets. Serum concentration of OVA-specific IgE (b), IgG2a (c), and IgG1 antibodies (d) was measured throughout the vaccination experiment by ELISA. *β*-Hexosaminidase release from RBL 2H3 cells upon crosslinking with OVA was performed with pooled sera from the final bleeding (e). Results are means ± SD from five mice per group (b, c, d) or means of three technical replicates measured using the same serum pool to sensitize the RBL 2H3 cells (e). ^∗^*P* < 0.05; ^∗∗^*P* < 0.01; ^∗∗∗^*P* < 0.001.

Finally, because of the promising results obtained for the immune-activating capacities of CL531, in combination with the lack of ERK1/2 phosphorylation observed in BMCMCs stimulated with CL531, we decided to investigate whether CL531 might suppress allergic sensitization in a prophylactic vaccination approach using an established mouse model of OVA-induced intestinal allergy (Figure 5(a), for more information on the experimental groups see Repository Figure 5) [22].

We found that intranasal coapplication of CL531 and OVA, but not CL531 or OVA alone, resulted in significantly reduced OVA-specific IgE levels after 7 days of challenge with OVA-containing food pellets, compared to vaccination with OVA alone (Figure 5(b)). OVA-specific IgE levels were also lower in the mice vaccinated with CL531 and OVA compared to those vaccinated with PBS, although this difference was not statistically significant (*p* = 0.215). Vaccination with CL531 plus OVA strongly enhanced OVA-specific IgG1 titers compared to nonvaccinated animals (Figure 5(c)). Moreover, no significant differences in OVA-specific IgG1 levels were observed among animals vaccinated with the dual ligand (CL531) alone, CL531 with OVA, or with Pam_2_CysK_4_ plus OVA. In addition, animals vaccinated with the mixture of CL531 and OVA displayed significantly increased levels of OVA-specific IgG2a antibodies compared to animals vaccinated with PBS or with either OVA alone or CL264 plus OVA (Figure 5(d)). Similar levels of OVA-specific IgG2a antibodies were observed in animals vaccinated with CL531 plus OVA, CL531 alone, and Pam_2_CysK_4_ plus OVA, suggesting that IgG2a production was driven by TLR2-signaling induced by both CL531 and Pam_2_CysK_4_.

In line with the results obtained for OVA-specific IgE levels, RBL 2H3 cells passively sensitized with pooled serum from animals vaccinated with CL531 plus OVA displayed the lowest levels of antigen- (OVA-) induced *β*-hexosaminidase release, compared to all other experimental groups (Figure 5(e) and Repository Figure 6). Here, vaccination with CL531 plus OVA significantly reduced mediator release from RBL-2H3 cells compared to PBS-vaccinated and OVA-sensitized animals (PBS group). By contrast, vaccination with OVA alone, significantly increased *β*-hexosaminidase release compared to nonvaccinated animals (PBS group, Figure 5(e) and Repository Figure 6). Vaccination with CL264 plus OVA or Pam_2_CysK_4_ plus OVA also resulted in higher levels of *β*-hexosaminidase release compared to nonvaccinated animals (Figure 5(e) and Repository Figure 6).

## 4. Discussion

Currently, TLR2 ligands are undergoing clinical testing as adjuvants in vaccines for the treatment of Lyme disease, malaria, HIV, HBV, and HPV, while TLR7 ligands are being tested for their benefits in the treatment of cancer (leukemia, prostate cancer, melanomas, breast cancer, and B cell lymphomas), chronic hepatitis, asthma, and rhinitis [2–4, 23]. TLR2 ligands, like the synthetic Pam_3_CysK_4_, have repeatedly been shown to induce tolerogenic DC and Tr responses [8, 9, 11], and TLR7 ligands show promising adjuvant capacity but are limited in their usage due to their induction of proinflammatory cytokines and the associated side effects [5, 6]. In contrast, several reports suggest that TLR7 ligands have potential as adjuvants for the treatment of Th2-mediated allergic diseases: in an experimental mouse allergy model, the TLR7 ligand Imiquimod significantly inhibited chronic inflammation, persistent airway hyperreactivity (AHR), and airway remodeling, while reducing serum IgE levels and Th2 cytokines in BAL fluids [24]. In addition, the Imiquimod-derivative resiquimod (R848) was shown to inhibit IgE production and induce IFN-*γ* secretion *in vivo* [25]. In line with these findings, prophylactic, epicutaneous treatment with R848 and the major birch pollen allergen Bet v 1 inhibited the production of biologically active Bet v 1-specific IgE antibodies, suppressed lung inflammation, and reduced AHR compared to Bet v 1 applied alone [26]. Finally, in a mouse model of established, OVA-induced allergic asthma, therapeutic treatment with R848 reduced allergic features via a Tr-dependent mechanism [27]. However, until now, no data have been available describing the application of the TLR7 activator CL264 as adjuvant for the treatment of allergic diseases.

In light of the studies reviewed above and the results presented in this study, we hypothesize that the dual TLR2/7 ligands CL413 and CL531, but not CL401, hold potential as adjuvants for the treatment of Th2-mediated allergic diseases. Upon stimulation with CL413 and CL531, we observed a strong anti-inflammatory IL-10 secretion from BMmDCs, correlating with a suppression of OVA-induced IL-5 and IL-13 secretion.

Moreover, direct mast cell activation by these TLR2/7 ligands was rather limited and we observed a suppression of DNP-HSA-induced, IgE-and-Ag-specific mast cell degranulation *in vitro* as well as the production of Th1-promoting IL-12 secretion upon *in vivo* application of these TLR2/7 ligands to naïve mice. In the context of allergy treatment, the immune-modulating effects of all three dual TLR2/7 ligands on mast cells are of special interest, since they constitute evidence of potentially important safety features. Moreover, in contrast to CL413, mast cell stimulation with CL401 or CL531 did not result in ERK1/2 phosphorylation, which is known to be associated with mast cell activation.

Interestingly, although structurally very similar (Repository Figure 1), the different dual TLR2/7 ligands displayed clearly distinct immune activation profiles. CL413 and CL531 induced epithelial cell-derived chemoattractant CCL2 secretion and BMmDC-derived IL-10 secretion, suppressed both OVA-induced IL-5 secretion and Ag-specific mast cell degranulation, and induced proinflammatory cytokine secretion *in vivo*. In contrast to this, CL401 induced considerably less IL-10 secretion from BMmDCs, induced IL-17A production in BMmDC:TC cocultures, and failed to induce either C57BL/6 BMCMC IL-6 secretion *in vitro* or IL-6 and TNF-*α* production *in vivo*. Among these findings, the IL-17A production observed in BMmDC:TC cocultures costimulated with CL401 and OVA is especially important. Indeed, concerns regarding the skewing of T cell responses towards Th17-dominated responses by the TLR2 ligand lipoprotein OspA, and the potential for this to contribute to the development of autoimmune diseases, have stopped the commercialization of a Lyme disease vaccine containing this TLR2 ligand [28].

Of note, among the tested TLR ligands, only *in vivo* application of CL413 resulted in a robust, and compared to the other groups, highly significant secretion of IFN-*α* (Figure 4). This result suggests that only CL413 may be able to activate plasmacytoid DCs. However, we cannot exclude the possibility that the increased secretion of either IL-6 or IL-10 from Flt-3L cultures upon stimulation with either CL531 or CL413 may in part be mediated by plasmacytoid DCs within these cultures (Repository Figure 3). Here, interestingly, both CL413 and CL531 induced significantly higher IL-6 and tended to induce higher (but not statistically significant) IL-10 secretion than observed for CL401. Since IFN-*α* is known to induce flu-like symptoms such as fever, fatigue, and joint pain [29], we suggest that the observed immune-activating profile of CL531, combined with a lack of IFN-*α* induction (as observed for CL531, but not for CL413), is likely to prevent these side effects observed upon *in vivo* application of such adjuvants, particularly CL531.

Indeed, in our own *in vivo* prophylactic treatment experiment with the dual TLR2/7 ligand CL531 *in vivo*, we demonstrated that coapplication of CL531 with OVA as a vaccine, but not vaccination with CL531 or OVA alone, displayed promising immune-modulating effects, suppressing OVA-specific IgE antibody production, in comparison to mice vaccinated with OVA alone while increasing the production of potentially blocking OVA-specific IgG1 and IgG2a antibodies *in vivo*. In line with these results, sera from animals vaccinated with the mixture of CL531 plus OVA showed the lowest capacity to sensitize RBL-2H3 cells for antigen-dependent degranulation *in vitro*. These results are in agreement with our *in vitro* results, where CL531 or CL413 induced by themselves only limited mast cell activation but suppressed DNP-HSA-induced, IgE-and-Ag-specific mast cell degranulation.

Given the way we tested our serum's ability to passively sensitize RBL-2H3 cells to respond to OVA challenge *in vitro* (which involved washing the cells after incubating them with sera for 1 h), we were more likely to detect the presence of anti-OVA IgE than anti-OVA IgG1 antibodies. In line with this, Segal and coworkers reported that although IgG receptors can be detected on RBL-2H3 cells, their affinity for IgG is much lower than the affinity of IgE for Fc*ε*RI [30]. Moreover, IgG aggregates bound to RBL-2H3 cells were shown to neither elicit mediator release by themselves nor modulate IgE-mediated histamine release [30]. However, we cannot exclude the possibility that some of the OVA-specific IgG1 antibodies which were induced might be able to activate mast cells *in vivo* via an IgG1-antigen immune complex-dependent mechanism [31], as OVA-specific IgG1 antibodies were increased in sera of animals vaccinated with CL531 plus OVA (Figure 5).

Notably, clinical trials of allergen-specific immunotherapy in humans however suggest that a successful therapeutic outcome can be achieved in association with the induction of blocking allergen-specific IgG4 antibodies even when allergen-specific IgG1 and IgE levels remain unchanged over long periods of time [32]. The serology of animals vaccinated with CL531 and OVA thus mirrored these results observed in human trials regarding the induction of allergen-specific IgG1 and IgG2a antibodies, while also significantly reducing levels of allergen-specific IgE.

Taken together, our results indicate that *in vivo*, CL531, coadministered with OVA, shows promising immune-modulating effects, suppressing the main features of allergy: allergen-specific IgE production and IgE-dependent mast cell degranulation and mast cell activation, while inducing the production of allergen-specific IgG antibodies. We speculate that these effects may reflect, at least in part, the ability of these dual TLR2/7 ligands to induce IL-10 secretion from BMmDCs.

These results are in line with the recently reported study by Gutjahr et al. who showed that the structurally very similar dual TLR2/7 ligand PamadiFectin (CL307) induced significantly stronger human DC and cytotoxic T cell responses than the nonconjugated TLR2- and TLR-7 ligands, provided either alone or as a mixture [16]. In accordance with our own results, application of CL307 resulted in highly enhanced production of antigen-specific IgG2a antibodies against the coapplied HIV antigen p24 [16].

The observed difference in immune-modulating capacity between the different dual TLR2/7 ligands might be explained by sterical hindrance of ligand binding to TLR2 and TLR7 induced by the conjugation. Furthermore, the lack of the Pam_2_CysK_4_-associated four lysine residues in CL401 in comparison to unconjugated Pam_2_CysK_4_ is likely to result in reduced and/or altered TLR2 activation capacity. In line with this hypothesis, when the capacities of the different TLR2 and TLR7 ligands to activate their target receptors were determined using TLR2- or TLR7-transfected HEK293 cells, Pam_2_CysK_4_ and CL531 were shown to induce identical levels of TLR2 activation, whereas CL413 and CL401 were 10- and 100-fold weaker TLR2 activators, respectively [15]. Correspondingly, TLR7 activation in transgenic HEK293 cells was shown to be identical for CL401, CL413, and CL531, with all dual TLR2/7 ligands being 10-fold weaker TLR7 activators compared to the unconjugated TLR7 ligand CL264 [15].

It is interesting that the immune-modulating effects could not be achieved when using the individual TLR2 and TLR7 ligands alone or as a simple mixture of these ligands. Therefore, we think that the combination of TLR2- and TLR7 ligands into dual TLR2/7 ligands is a promising strategy to leverage the beneficial immune-modulating properties of each of the single ligands while also ensuring the simultaneous costimulation of the target cell with both components in a fixed one-to-one ratio. Moreover, TLR2-mediated uptake of the dual TLR2/7 ligands is likely to facilitate intracellular delivery to TLR7 and therefore enhance immune cell activation and subsequent immune responses. We hypothesize that the 3-dimensional structure of dual TLR2/7 ligands is a key factor for their immunomodulatory effect. Our results suggest that CL413 and CL531, but not CL401, have a structure that allows the interaction between the TLR2-activating Pam_2_CysK_4_ component of the dual ligand with TLR2 on the cell surface. This interaction likely has two main effects: (1) the induction of immunosuppressive IL-10 secretion by DCs via TLR2 activation in agreement with previous studies [8, 33] and (2) internalization of the dual ligand, facilitating the interaction of the contained TLR7 ligand with TLR7, contributing to the induction of Th1-biassed immune responses by synergic activation of signaling cascades leading to IL-10 gene expression. In line with this hypothesis, we observed that the dual TLR2/7 ligands retained the ability to induce both TLR2- and TLR7-mediated cytokine secretion upon *in vivo* application (Figure 4).

In accord with this speculation, stimulation of DCs with Pam_3_CysK_4_ chemically conjugated to an OVA-derived CD8^+^ TC peptide resulted in enhanced uptake and presentation of the OVA-derived CD8^+^ TC peptide by the stimulated DCs [12].

Considering the overall immune-modulating properties of the different ligands, we think that dual TLR2/7 ligands have the potential to induce Th1-biased immune modulation *in vivo*. Therefore, we suggest that the dual TLR2/7 ligands CL413 (with the caveat of its high IFN-*α* induction *in vivo*) and especially CL531, due to its reduced ability to activate mast cells *in vitro* and capacity to suppress allergen-specific IgE production *in vivo* (together with a lower capacity than CL413 to induce IFN-*α*), are promising adjuvant candidates to further improve the treatment of allergic diseases. These findings also suggest that it will be of substantial interest to conduct future studies to elucidate the molecular mechanisms by which these promising adjuvants can modulate immune responses.

## Supplementary Material

Repository figure 1: Chemical structures of single (Pam_2_CysK_4_ and CL264, A) and dual (CL401, CL413, and CL431, B) TLR-ligands used in this study. Repository Fig. 2: Gating strategies and phenotypes of bone marrow-derived myeloid and plasmacytoid dendritic cells used for this study. Gating strategies for bone marrow-derived myeloid (A, BMmDC, CD11b^+^CD11c^+^B220^−^) and plasmacytoid (B, BMpDC, CD11b^−^CD11c^+^B220^+^) dendritic cells. The upper row demonstrates the gating strategy, whereas the lower row shows the expression levels of the indicated surface markers in ungated mDC and pDC cultures on day 8 of differentiation, respectively. For the identification of CD11b^+^CD11c^+^B220^−^ BMmDCs GM-CSF-cultures were first gated on B220^−^CD11c^+^ cells (indicated by the red box), BMmDCs were then identified within this gate as CD11c^+^CD11b^+^ double positive cells (A). For the identification of CD11b^−^CD11c^+^B220^+^ BMpDCs Flt-3L-cultures were first gated on B220^+^CD11c^+^ cells (indicated by the red box), BMpDCs were then identified within this gate as CD11c^+^CD11b^−^ cells (B). Repository Fig. 3: The dual TLR2/7-ligands CL413 and CL531 also activate dendritic cells in Flt-3L-cultures. Cytokine secretion from BALB/c-derived Flt-3L-cultures stimulated as indicated after 8 days of differentiation. Data are mean results of three independent experiments ± SD. Repository figure 4: CL413 and CL531, but not CL401 or CL264, induce levels of CCL2 secretion from mouse epithelial cells similar to that induced by Pam_2_CysK_4_. CCL2 secretion from LA-4 epithelial cells stimulated for 24 h with equimolar amounts of the different TLR2/7-ligands. Data are mean results of two independent experiments ±SD. Repository figure 5: Experimental groups in prophylactic vaccination experiment. Repository figure 6: Sera obtained after prophylactic vaccination with CL531 induce reduced mediator release from RBL 2H3 cells upon crosslinking with OVA. β-hexosaminidase release from RBL 2H3 cells upon crosslinking with OVA was performed with sera from the final bleed. Results are means of three technical replicates measured using the same serum pool. Indicated are the statistical differences from the PBS group for the highest stimulation concentration (10 μg/mL OVA).

## Abbreviations

BMmDCs: Bone marrow-derived myeloid dendritic cells
BMpDCs: Bone marrow-derived plasmacytoid dendritic cells
BMCMCs: Bone marrow-derived cultured mast cells
MCs: Mast cells
OVA: Ovalbumin
PAMPs: Pathogen-associated molecular patterns
TCs: T cells
TLR: “Toll-” like receptor
Th: T helper cells
Tr: T regulatory cells.
