# Supplementary material for: Targeting of Immune Cells by Dual TLR2/7 Ligands Suppresses Features of Allergic Th2 Immune Responses in Mice
Source: J Immunol Res. 2017 Oct 24;2017:7983217. doi: 10.1155/2017/7983217 (PMC5674512; doi:10.1155/2017/7983217)
Supplement: Supplementary file 1 — Repository figure 1: Chemical structures of single (Pam2CysK4 and CL264, A) and dual (CL401, CL413, and CL431, B) TLR-ligands used in this study. Repository Fig. 2: Gating strategies and phenotypes of bone marrow-derived myeloid and plasmacytoid dendritic cells used for this study. Gating strategies for bone marrow-derived myeloid (A, BMmDC, CD11b+CD11c+B220−) and plasmacytoid (B, BMpDC, CD11b−CD11c+B220+) dendritic cells. The upper row demonstrates the gating strategy, whereas the lower row shows the expression levels of the indicated surface markers in ungated mDC and pDC cultures on day 8 of differentiation, respectively. For the identification of CD11b+CD11c+B220− BMmDCs GM-CSF-cultures were first gated on B220−CD11c+ cells (indicated by the red box), BMmDCs were then identified within this gate as CD11c+CD11b+ double positive cells (A). For the identification of CD11b−CD11c+B220+ BMpDCs Flt-3L-cultures were first gated on B220+CD11c+ cells (indicated by the red box), BMpDCs were then identified within this gate as CD11c+CD11b− cells (B). Repository Fig. 3: The dual TLR2/7-ligands CL413 and CL531 also activate dendritic cells in Flt-3L-cultures. Cytokine secretion from BALB/c-derived Flt-3L-cultures stimulated as indicated after 8 days of differentiation. Data are mean results of three independent experiments ± SD. Repository figure 4: CL413 and CL531, but not CL401 or CL264, induce levels of CCL2 secretion from mouse epithelial cells similar to that induced by Pam2CysK4. CCL2 secretion from LA-4 epithelial cells stimulated for 24 h with equimolar amounts of the different TLR2/7-ligands. Data are mean results of two independent experiments ±SD. Repository figure 5: Experimental groups in prophylactic vaccination experiment. Repository figure 6: Sera obtained after prophylactic vaccination with CL531 induce reduced mediator release from RBL 2H3 cells upon crosslinking with OVA. β-hexosaminidase release from RBL 2H3 cells upon crosslinking with OVA was performed w [file 7983217.f1.pdf]

Targeting of immune cells by dual TLR2/7 ligands suppresses features of allergic T_H2 immune responses in mice

Laiño Jonathan[1], Wangorsch Andrea[1], Blanco Perez Frank[1], Wolfheimer Sonja[1], Krause Maren[1], Flaczyk Adam[1], Möller Tobias-Maximilian[1], Tsai Mindy[2], Galli Stephen[2], Vieths Stefan[1], Toda Masako[1], Scheurer Stephan[1], Schülke Stefan[1*]

[1]Paul-Ehrlich-Institut, Langen, Germany, Vice President´s Research Group 1: Molecular Allergology

[2]Stanford University School of Medicine, Stanford, USA, Department of Pathology and the Sean N. Parker Center for Allergy and Asthma Research

Short title: Dual TLR2/7-ligands as allergy adjuvants

*Corresponding author:

Stefan Schülke, PhD

Paul-Ehrlich-Institut, Vice President´s Research Group 1: Molecular Allergology

Paul-Ehrlich-Str. 51-59, 63225 Langen, Germany

phone: +49 6103 775209

fax: +49 6103 771258

stefan.schuelke@pei.de

Repository figures:

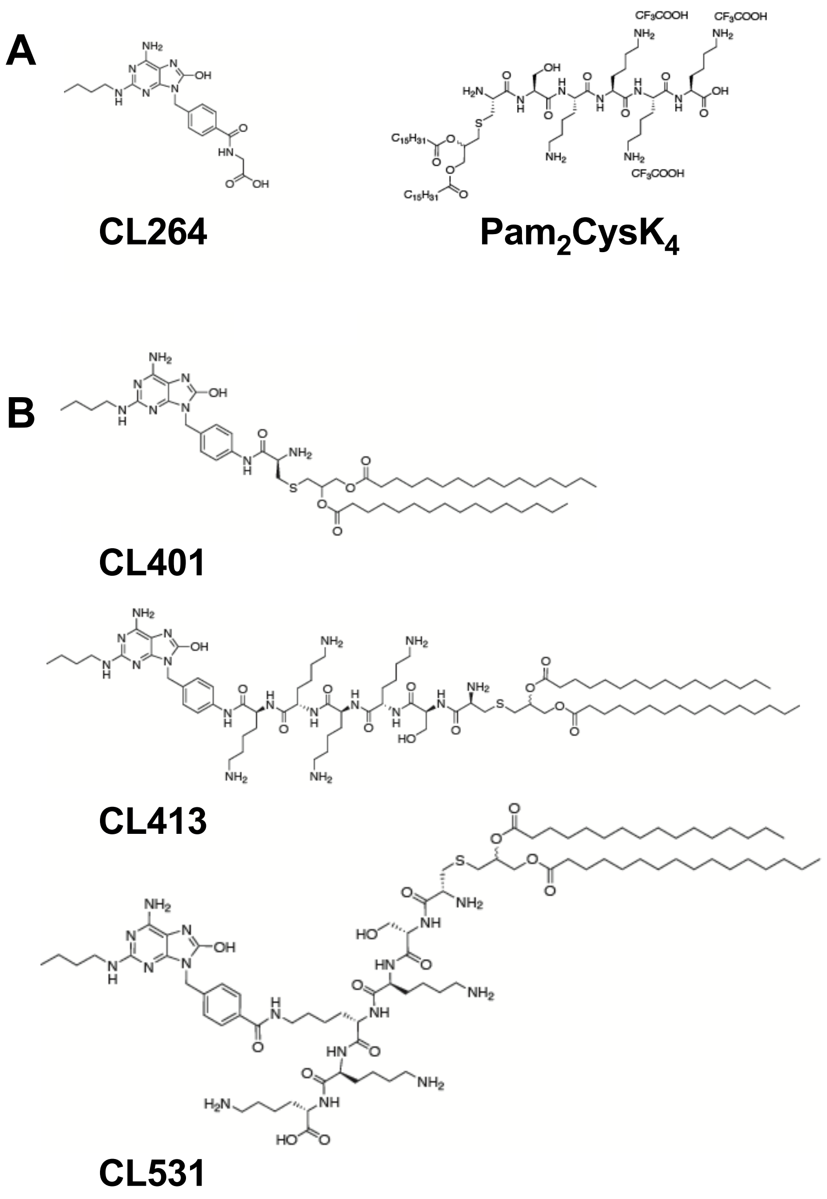

Repository figure 1: Chemical structures of single (Pam_2CysK_4 and CL264, A) and dual (CL401, CL413, and CL431, B) TLR-ligands used in this study.

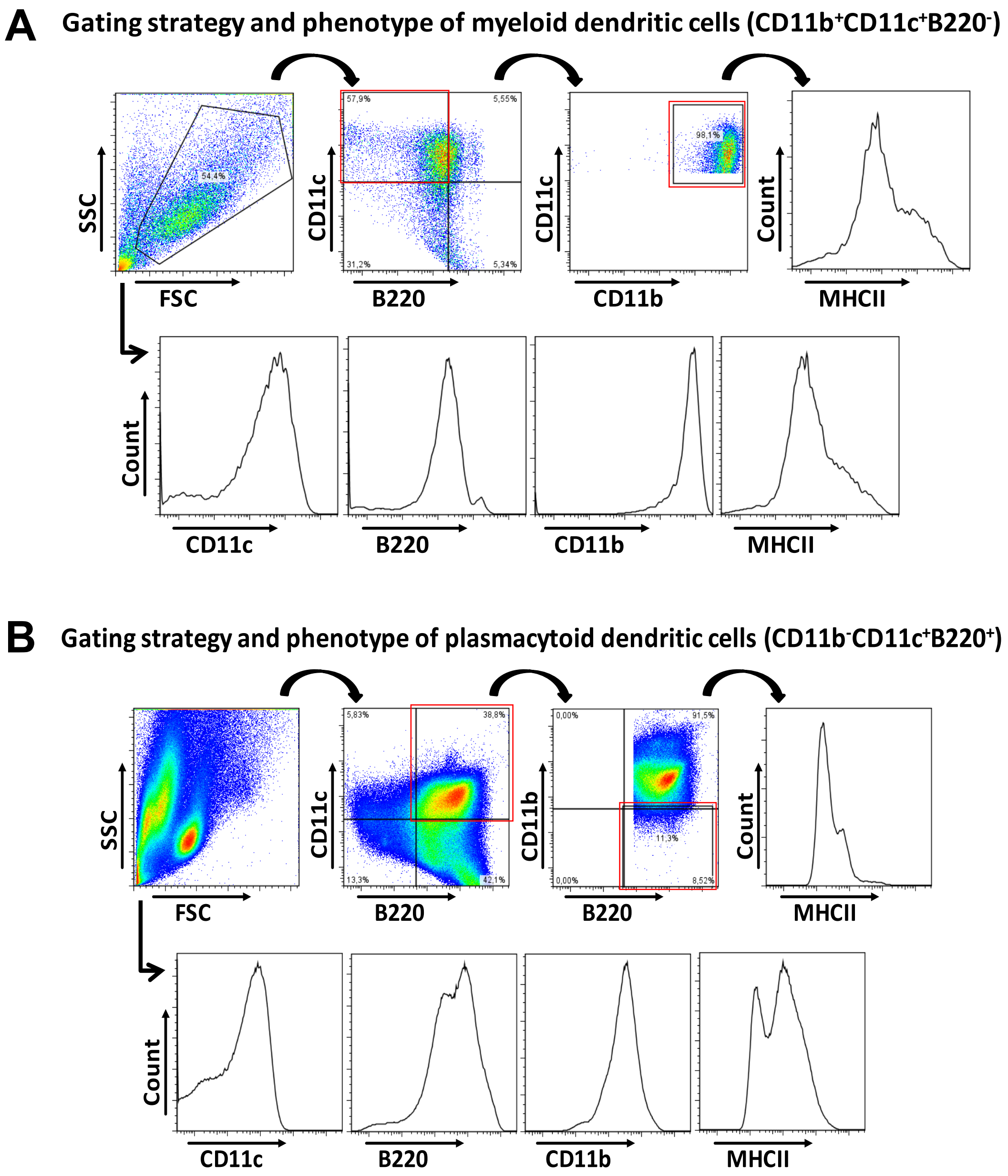

Repository Fig. 2: Gating strategies and phenotypes of bone marrow-derived myeloid and plasmacytoid dendritic cells used for this study. Gating strategies for bone marrow-derived myeloid (**A**, BMmDC, CD11b$^+$CD11c$^+$B220$^-$) and plasmacytoid (**B**, **BMpDC,** CD11b$^-$CD11c$^+$B220$^+$) dendritic cells. The upper row demonstrates the gating strategy, whereas the lower row shows the expression levels of the indicated surface markers in ungated mDC and pDC cultures on day 8 of differentiation, respectively. For the identification of CD11b$^+$CD11c$^+$B220$^-$ BMmDCs GM-CSF-cultures were first gated on B220$^-$CD11c$^+$ cells (indicated by the red box),

BMmDCs were then identified within this gate as CD11c⁺CD11b⁺ double positive cells (**A**). For the identification of CD11b⁻CD11c⁺B220⁺ BMpDCs Flt-3L-cultures were first gated on B220⁺CD11c⁺ cells (indicated by the red box), BMpDCs were then identified within this gate as CD11c⁺CD11b⁻ cells (**B**).

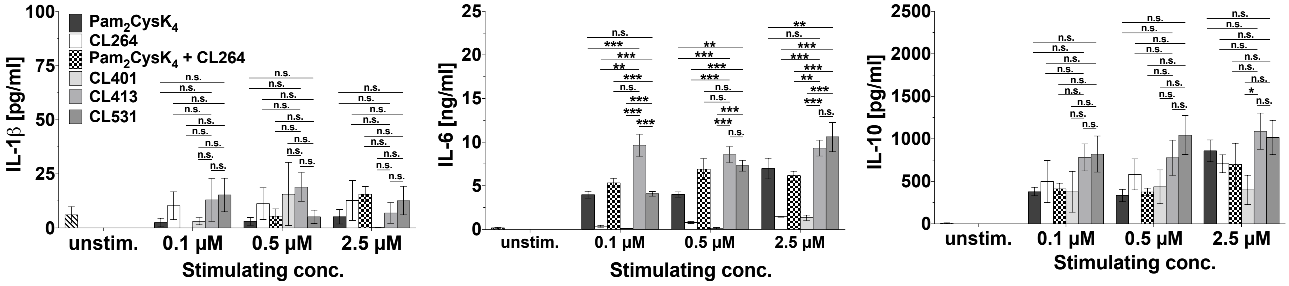

Repository Fig. 3: The dual TLR2/7-ligands CL413 and CL531 also activate dendritic cells in Flt-3L-cultures. Cytokine secretion from BALB/c-derived Flt-3L-cultures stimulated as indicated after 8 days of differentiation. Data are mean results of three independent experiments ± SD.

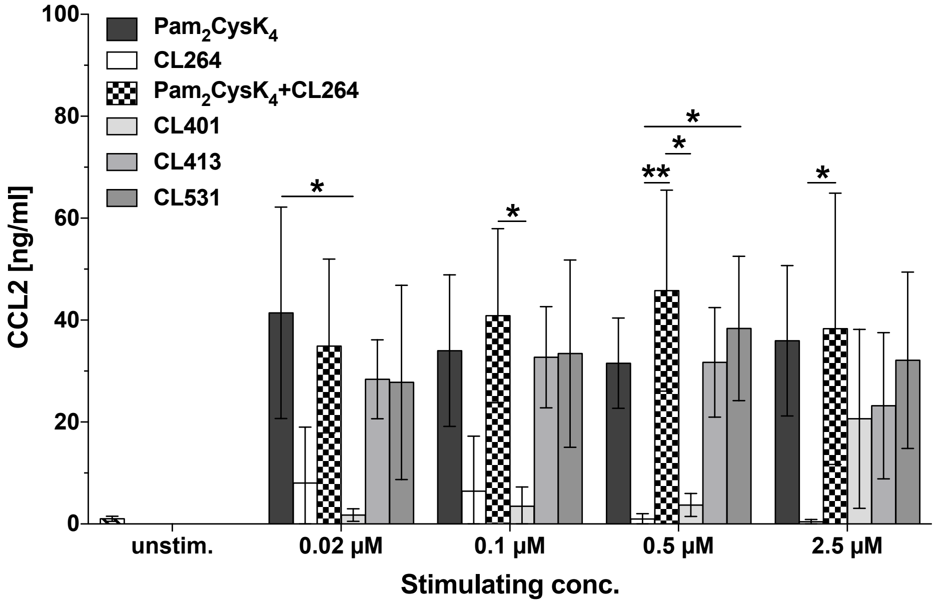

Repository figure 4: CL413 and CL531, but not CL401 or CL264, induce levels of CCL2 secretion from mouse epithelial cells similar to that induced by

Pam$_2$CysK$_4$. CCL2 secretion from LA-4 epithelial cells stimulated for 24 h with equimolar amounts of the different TLR2/7-ligands. Data are mean results of two independent experiments ±SD.

Group name	Vaccination (i.n.)	Sensitization (i.p.)	Challenge (via food pellets)
Mock	PBS	PBS	Conventional diet
PBS	PBS	2 x OVA (10 µg) + Alum (2 mg)	Egg white diet
OVA	rOVA (10 µg)	2 x OVA (10 µg) + Alum (2 mg)	Egg white diet
Pam$_2$CysK$_4$+OVA	Pam$_2$CysK$_4$ (0.5 mM) + rOVA (10 µg)	2 x OVA (10 µg) + Alum (2 mg)	Egg white diet
CL264+OVA	CL264 (0.5 mM) + rOVA (10 µg)	2 x OVA (10 µg) + Alum (2 mg)	Egg white diet
CL531	CL531 (0.5 mM)	2 x OVA (10 µg) + Alum (2 mg)	Egg white diet
CL531+OVA	CL531 (0.5 mM) + rOVA (10 µg)	2 x OVA (10 µg) + Alum (2 mg)	Egg white diet

Repository figure 5: Experimental groups in prophylactic vaccination experiment.

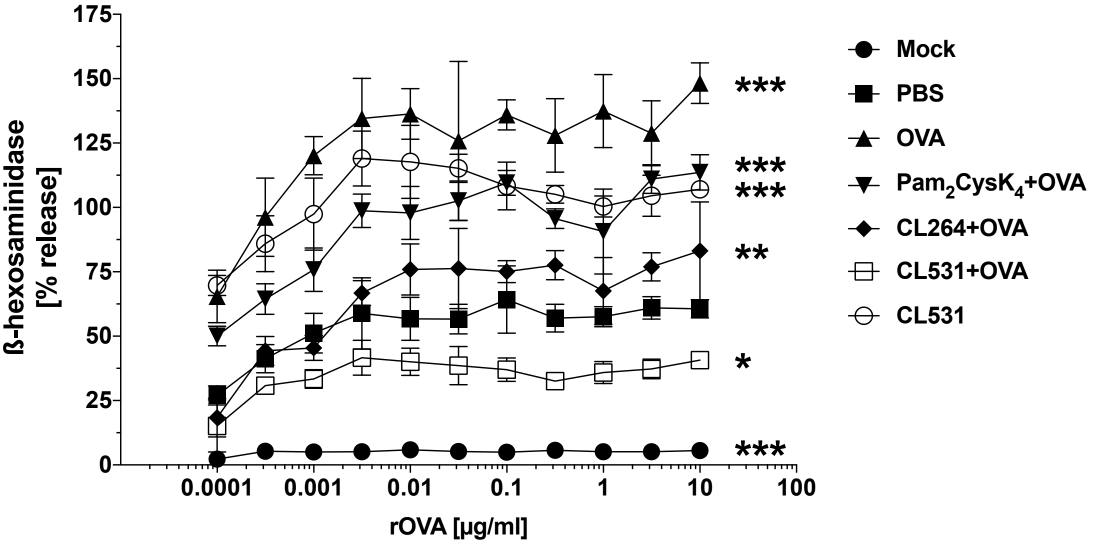

Repository figure 6: Sera obtained after prophylactic vaccination with CL531 induce reduced mediator release from RBL 2H3 cells upon crosslinking with

OVA. β-hexosaminidase release from RBL 2H3 cells upon crosslinking with OVA was performed with sera from the final bleed. Results are means of three technical replicates measured using the same serum pool. Indicated are the statistical differences from the PBS group for the highest stimulation concentration (10 μg/mL OVA).
